# Fertilizer and fungicide reduce herbicide efficacy and enhance growth of invasive common tansy (*Tanacetum vulgare*)

**DOI:** 10.1371/journal.pone.0333818

**Published:** 2025-10-22

**Authors:** Jonathan A. Bennett, Obaida Elshamy, Matthew Trefiak, John Paul Wasan

**Affiliations:** Department of Plant Sciences, University of Saskatchewan, Saskatoon, Saskatchewan, Canada; Universidade de Coimbra, PORTUGAL

## Abstract

Common tansy (*Tanacetum vulgare*; Asteraceae) is a widespread invasive species in North America that threatens biodiversity and agricultural productivity by displacing resident vegetation. Combined with being unpalatable, it can be toxic and thus poses significant challenges for the livestock industry. Current tansy control strategies are largely chemical and rely on a suite of synthetic auxin herbicides. The need for reapplication may lead to resistance development in addition to significant biodiversity losses. Recent work suggests that invasive Asteraceae may rely on symbiotic arbuscular mycorrhizal fungi (AMF) to give them a competitive advantage. We hypothesized that suppressing AMF would reduce tansy growth and reduce reliance on more damaging herbicides. Fungicides and fertilizers, known to suppress AMF, may be potential tools for tansy suppression by reducing its competitive ability; however, both may also enhance invader growth and represent a significant risk. We conducted a two-year experiment crossing three herbicides, with varying degrees of residual control, with fungicide and fertilizer treatments to explore their effects on tansy. Despite initially reducing AMF abundances, both fertilizer and fungicide unexpectedly improved tansy growth, especially when applied with the non-residual herbicide (2,4-D), where strong control was eliminated by either treatment. This suggests that, at least at our study site, any suppression of AMF did not affect tansy strongly enough to overcome the benefits of increased nutrients and pathogen suppression. Independent of fungicide or fertilizer, all three herbicides reduced tansy biomass and increased community biomass by year two, driven by increases in grasses. The most effective herbicide (picloram), however, also caused the greatest declines in broadleaf plants, leading to significant species losses. Conversely, 2,4-D was only slightly less effective after two years, while having limited non-target effects. Non-residual herbicides, like 2-4D, may offer a better balance between tansy control and biodiversity conservation.

## Introduction

Invasive plants are a large ecological and economic problem, causing a loss of species, reduced ecosystem services, and damage to infrastructure [1,2], thus costing the global economy billions annually [3–5]. Herbicides are often the most effective means of control but, especially for perennial broadleaf species, herbicide resistance may develop as most common herbicides have similar modes of action [6,7]. The development of herbicide resistance is even more likely as the same or similar herbicides are frequently reapplied to maintain control, thus strengthening the selection on herbicide resistance [8,9]. To maintain herbicide efficacy, it is critical to find ways of augmenting control and reducing the frequency of herbicide application.

Common tansy (*Tanacetum vulgare*; Asteraceae) originated in Europe where it is common to riparian areas and abandoned fields, but is now invasive throughout much of North America where it is found in disturbed areas and grasslands [10–12]. It is a tall, broadleaf plant that can be highly competitive [13]. Multiple herbicides can provide effective control but can have non-target effects [14,15]. Herbicide efficacy and non-target effects are typically limited to the time the chemical can remain active before degradation (i.e., the longevity of the residual effect) [10,11], representing a trade-off between control and non-target effects. Mechanical control can be effective in limiting seed set, but it can quickly recover from mowing, meaning repeated management is required [10,13]. Common tansy also produces many compounds that limit grazing, although sheep will consume tansy with unknown risks to sheep reproduction [10,11]. Introduction of specialized herbivorous insects or pathogens is challenging due to high chemical diversity within and among invasive populations, which can limit the effectiveness of specialist natural enemies [16–18]. Consequently, alternative strategies are required to effectively manage this invader.

Many invasive Asteraceae are highly reliant on symbiotic arbuscular mycorrhizal fungi (AMF) that provide them with nutrients and improve pathogen resistance [19,20]. Disrupting this mutualism may improve control of these plants. Certain fungicides can be used to suppress AMF, which can reduce the abundance of AMF dependent species [21,22]. Fungicides, however, have been developed to control pathogens rather than suppress AMF, and can thus improve growth of non-AMF dependent plants in natural grassland ecosystems [23]. Nutrient-rich environments can also reduce the benefits of AMF by reducing plant need for AMF-derived nutrients [24]. By altering trade between plants and AMF, fertilizer addition can thus disrupt mycorrhizal symbioses [25], suggesting that fertilizers may suppress AMF dependent invaders [26]. Moreover, fertilizers can improve the competitive ability of resident grasses, which can reduce recovery of the invasive species from herbicide applications [27], provided the invader is not a better competitor for those nutrients.

Within its native range, tansy is extensively colonized by AMF, can promote other symbiotic fungi (dark septate endophytes), and benefits from both these associations [28–31]. Further, tansy can take advantage of symbiotic fungi associated with other species [28], suggesting that it is capable of utilizing AMF within the invaded range. Data on tansy responses to AMF are lacking outside its native range. The limited data on plant-soil interactions in the invaded range, suggests that tansy responses to tansy conditioned soils (i.e., plant-soil feedbacks) are negative [17]; however, negative feedbacks are within the range of responses found within the native range [17,28]. Consequently, we hypothesized that tansy, like many invasive Asteraceae [19,20], would have positive responses to AMF in the invaded range and that AMF suppression would reduce tansy growth. Further, as grassland plants vary in their response to AMF [32,33], we also hypothesized that the risks to biodiversity may be lessened by suppressing AMF than by applying herbicides, especially those with longer residuals.

Whether tansy will benefit from or be controlled by fertilizer application is unclear. Tansy growth responses to fertilization are generally positive [34], potentially owing to its dense root system concentrated in the upper 60 cm of soil [10]. As many invaders, including tansy, are disturbance adapted [10,12], it is unclear if tansy can compete with established grassland vegetation in absence of disturbance, given that grasses are often highly competitive for soil resources and increase when grasslands are fertilized [33]. Tansy, however, can suppress some native species [13] and can form monocultures when it invades [10], indicating some competitive capacity. Further, fertilizers can cause significant losses of grassland plant diversity [35]. Combined this represents significant risk associated with fertilizing tansy invaded areas.

To determine whether AMF suppression could improve tansy control, we conducted a two-year factorial field experiment in a diverse seeded pasture. The experiment crossed four herbicide treatments (control, 2,4-D, picloram, and aminocyclopyrachlor [ACP] + metsulfuron [MS]) with three AMF suppression treatments (control, fungicide [thiophanate-methyl], and fertilizer [urea + triple super phosphate]). All three herbicides are registered for control of common tansy in Canada and are commonly used by local livestock producers. We included three herbicides as we wanted to compare commonly used residual herbicides (picloram and ACP + MS) that differ in the amount of time they remain active [picloram has longer residual effects; 36], with a non-residual herbicide (2,4-D) as we hypothesized that shorter residuals may reduce non-target effects. For two years, we monitored treatment effects on AMF colonization, tansy biomass, community biomass, functional group cover, and plant species richness. We hypothesized that: 1) both fungicide and fertilizer would reduce AMF colonization and thus reduce common tansy by limiting its competitive ability; 2) the residual herbicides (picloram and ACP + MS) would provide strong control of tansy and increase grass biomass, but that 2,4-D, which lacks a residual, would only provide benefits in the first year; 3) fungicide and fertilizer would enhance 2,4-D benefits to be equivalent to the residual herbicides; and 4) 2,4-D plus fungicide would have the smallest impact on broadleaf plants and species richness, but that fertilizer would reduce plant richness regardless of the herbicide treatment.

## Materials and methods

### Study site

The research took place at the University of Saskatchewan Livestock and Forage Centre of Excellence’s Pathlow Research Pasture, located in the Boreal Transition eco-region of the Northern Great Plains near Melfort, Saskatchewan (52.69°N, 104.97°W). The soils are Orthic Black Chernozems, rich in clay with glacial till parent material. The location chosen for the experiment was a grassy area surrounded by stands of trembling aspen (*Populus tremuloides)*. This area was previously seeded to a variety of agronomic grasses and legumes, with the dominant species being smooth brome (*Bromus inermis)*, Kentucky Bluegrass (*Poa pratensis)*, and cicer milkvetch (*Astragalus cicer*); however, native species now comprise approximately 20% of the vegetation. The area, and the pasture in general, have been heavily invaded by common tansy which represents more than 30% of total vegetative cover.

### Experimental design

**Fig 1:**
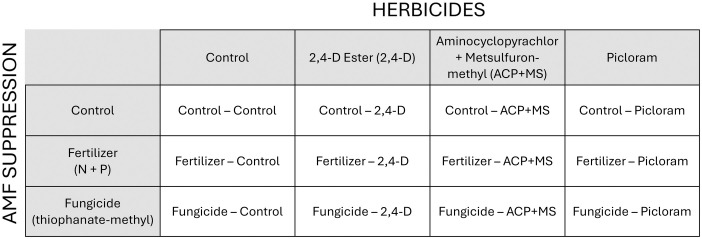
Factorial design of herbicide and arbuscular mycorrhizal fungi (AMF) suppression treatments used in the experiment.

Using a fully factorial randomized complete block design (12 treatments total), we tested whether manipulating AMF (three levels) could enhance the effect of herbicides (four levels) on common tansy (Fig 1). Treatment application began in mid-June 2022. For AMF manipulations, we included an untreated control, a fungicide containing thiophanate methyl shown to suppress AMF [21], and a fertilizer treatment. We also included three herbicides: 2,4-D (2,4-D Ester 700 [660 g acid equivalent (a.e.) L^-1^], Nufarm Canada, 333–96th Ave NE, Calgary, Alberta, Canada, T3K 0S3), picloram (Tordon^TM^ 22K [240 g picloram L^-1^], Corteva AgriScience, 115 Quarry Park Rd. SE, Calgary, AB, Canada, T2C 5G9), and aminocyclopyrachlor + metsulfuron-methyl (hereafter ACP + MS, TruRange [39.5% ACP, 12.6% MS], Envu Environmental Science, 137 Glasgow Street, Kitchener, Ontario, Canada, N2G 4X8) and a control. Both picloram and ACP + MS are residual herbicides that can provide effective multi-year tansy control, with picloram providing longer term control [36]. 2,4-D can also be effective but allows rapid recovery as it lacks a soil residual. The inclusion of a non-residual herbicide allows us to better evaluate whether suppressing AMF can reduce recovery from herbicide over the time frame of this study (two years). With the factorial design, there were 12 plots per block, with blocks replicated five times.

Fertilizers were applied twice and fungicide was applied three times. We applied the fertilizer at a rate of 60 kg/ha P (triple super phosphate) and 50 kg/ha N (urea) as both N and P fertilizers can reduce AMF benefits [37]. Fertilizer applications occurred in mid-June in both 2022 and 2023. After applying the fertilizers, we applied the wettable powder fungicide (Senator® 70WP, 70% thiophanate-methyl, Nippon Soda Co.) at a rate of 70 kg active ingredient (a.i.) in 2500 L ha^-1^ of water using a handpump sprayer. In addition to the mid-June applications, we also applied the fungicide in mid-July 2022. We did not apply the fungicide in mid-July 2023 as the experiment was ending within two-weeks, and we expected limited effect. Fungicide applications were also timed to be just prior to rainfall to improve soil penetration. Previous work using this fungicide to suppress AMF used a lower rate (12.5 kg a.i. h^-1^), but also applied the fungicide more frequently (every three weeks over a six month growing season) [21] than we did (twice over a five month growing season), resulting in similar amounts of fungicide being applied. The decision to increase the rate and reduce the frequency came as frequent fungicide application is impractical for land managers. We recognize, however, that fungicides can have multiple non-target effects on the plant-soil system that we did not quantify [38] and that the increased dosage may have amplified these effects.

Immediately following application of the initial AMF manipulation treatments, we applied the herbicides (mid June 2022; prior to flowering but with high vegetative biomass). Picloram was applied at 1.1 kg a.i. in 180 L of water ha^-1^. ACP + MS were applied with at 66 g a.i. ha^-1^ and 21 g a.i. ha^-1^, respectively, in 200 L of water ha^-1^. Following the manufacturers recommendation we also include 1% v/v Merge® surfactant (BASF Canada) with the ACP + MS application, which is intended to increase herbicide uptake via increased adherence and evenness of spray coverage. 2,4-D was applied at 2.2 kg a.e. in 100 L of water ha^-1^. All herbicides were applied to individual plots using a battery-operated backpack sprayer, with separate sprayers for each herbicide.

### Data collection

Species-level cover estimates were collected at the end of July in 2022 and 2023, using a 0.25 m^2^ square quadrat. We then grouped species into four functional groups: tansy, grasses, legumes, and other broadleaf plants. As we were interested in changes in the relative abundance of these groups, we divided their percent cover by the total percent cover for the plot. After the cover estimates, we also collected plant biomass samples by clipping a 0.1 m^2^ area to 1 cm stubble height. These samples were sorted into tansy and the remaining community, dried at 55°C for at least 48 h, then weighed.

At the same time as we collected the biomass samples, we collected samples for assessment of mycorrhizal colonization. The methods differed between the two years because, although we wanted to examine treatment effects on tansy colonization, there were too few tansy plants to directly collect tansy roots for colonization the year of herbicide application (2022), especially given that the required destructive sampling would influence data collection the following year. Instead, in 2022, we collected soil samples to assess colonization potential. Soils for colonization potential assessment were immediately placed on ice in the field, then refrigerated at 4°C until use. To assess colonization potential, we used a modified version of the methods from Moorman and Reeves [39], using sorghum sudangrass (*Sorghum × drummondii*) – another highly mycotrophic plant – rather than corn (*Zea mays*). We filled 50 ml centrifuge tubes with field soil, then seeded these tubes with sorghum. The tubes were then placed in a growth chamber with 16 h of light at a constant 24°C. Tubes were watered every 3 days for 24 days until harvest where we collected all root material. In 2023, as sufficient tansy had recovered, we collected root material from tansy plants for colonization assessments. Plants were excavated and a minimum of 50 cm of fine roots were collected. In both cases, roots were washed free of soil and stored in ethanol until colonization could be assessed. It is important to note that the two colonization assessments measure different things: the available of AMF propagules for plant colonization and the density of AMF within common tansy roots, which estimate tansy colonization by AMF over the longevity of the roots used, which may exceed the duration of the experiment.

Sample preparation for colonization assessment used a procedure modified from Pitet et al. [40]. Cleaned roots were first placed in tissue biopsy cassettes, then cleared by placing the cassettes in a 10% KOH (potassium hydroxide) solution at 95°C for 2 hours. We then rinsed the cassettes in tap water before a 30-second soak in 5% bleach solution to reduce external pigmentation. After bleaching, they were placed in 2% HCI (Hydrogen chloride) solution at 95°C for 30 minutes to acidify them. Acidified roots were stained using either a solution of acetoglycerol and 10% Schaeffer black ink in 2022 or 0.02% Trypan blue in 2023 at 95°C for 30 minutes. We initially used the ink; however, issues with stain adherence in other projects caused us to modify the procedure starting in 2023. After staining, the roots were rinsed and placed in weakly acidified water (distilled water plus a few drops of acetic acid) for at least 24 hours to remove excess stain. Approximately 10 cm of stained roots were then placed on microscope slide for colonization assessment.

The method of colonization assessment differed between years. As the roots were young for the 2022 colonization potential assessment, AMF structures were rare, so we looked for infection points (hyphae or spores penetrating the roots) and scored presence absence at 80 randomly selected points along the root using the line intercept method [41]. In 2023, we used the mature tansy roots and AMF structures were more prevalent, so we scored the presence of either vesicles or arbuscules across 50 points across the roots. Due to issues with root acquisition (sorghum mortality or no tansy in specific plots) and slide preparation, the total number of samples per year were reduced to 46/60 in 2022 and 31/60 in 2023. Chi squared tests indicated no biases in the distribution of samples among treatments (both P > 0.9); however, the reduction in sample size will reduce power in any associated statistical tests.

### Data analysis

To determine treatment effects on the response variables, we used a combination of linear and generalized linear mixed models in the lme4 package [42] in R v. 4.2.3. All code has been uploaded as S1 Appendix and all data as S2 Dataset. For the colonization assessment, we used generalized linear models with the Poisson distribution as these were count data. As the data collection differed between years, we ran separate models for infection points in 2022 and AMF structures in 2023. Both models included colonization as the response variable, with herbicide and AMF manipulation as factorial fixed effects and block as a random effect. To assess significance, we used Type III Wald chi-square tests in the ‘Anova’ function in the R package ‘car’ [43] after setting the contrasts to “contr.sum”.

For all remaining response variables, we used general linear mixed models with restricted maximum likelihood estimation. We included herbicide, AMF manipulation, and year as factorial fixed effects, to test how treatment effects varied over time due to variation in herbicide residuals. We also included block and plot nested within block as random effects to account for the spatial structure of the experiment and the repeated measures on each plot. After testing for normality and homogeneity of residuals using residual plots, Shapiro-Wilks tests, and Levene’s tests, we transformed the different response variables to minimize any violation of these assumptions: tansy biomass, community biomass, and legume cover were square-root-transformed and other broadleaf cover was log-transformed. For linear mixed models, model significance was assessed using F tests with Type III sums of squares in the ‘lmerTest’ package [44], with degrees of freedom assessed using Satterthwaite’s method. For both generalized and linear mixed models, model contrasts were compared to the sum across treatments (contrasts = “contr.sum”).

For all models, we conducted pairwise comparisons on significant terms using the emmeans package [45], with the Tukey method of adjustment for multiple comparisons. Due to the small sample size and the penalties associated with multiple comparisons, we note incidences of marginal effects (P <= 0.1) when interpreting the pairwise comparisons. When the interaction among treatments was significant, we did not compare all possible pairwise comparisons as many of those comparisons were not of interest. For herbicide *AMF suppression interactions, we ran two separate contrasts – among AMF treatments within each of the herbicides and among herbicides within the AMF treatments – to fully explore the dependency between the treatment groups. For herbicide * year and AMF * year interactions, we compared only the herbicide or AMF treatments within years as we were interested in how treatment effects differed between the first and second years.

## Results

**Fig 2:**
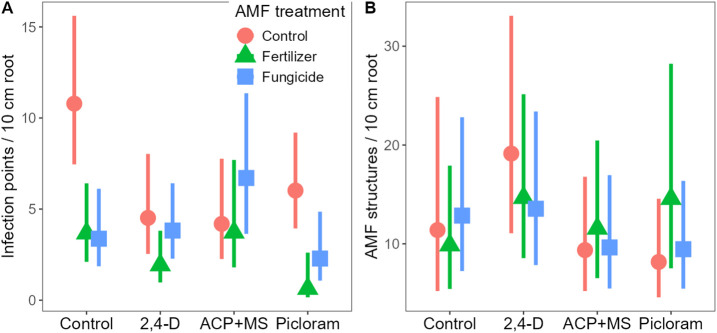
Herbicide and AMF manipulation effects on root colonization in 2022 (A) and 2023 (B). In 2022, colonization was assessed using a rapid phytometer assay of infection using field collected soils. In 2023, colonization was assessed using roots of excavated tansy plants. Shown are back-transformed model estimated marginal means and 95% confidence intervals. AMF manipulation treatments were dependent on herbicide treatment in 2022 (P = 0.010) but had no effect in 2023 (main effect P = 0.615, interaction P = 0.123).

Herbicides (Χ^2 ^= 18.28, d.f. = 3, P < 0.001), AMF manipulation (Χ^2 ^= 25.36, d.f. = 2, P < 0.001), and their interaction (Χ^2 ^= 16.84, d.f. = 6, P = 0.010) all had significant effects on colonization potential in 2022. All herbicides and AMF manipulation treatments caused declines in colonization potential; however, the effects varied among treatment combinations (Fig 2A). There was no effect of AMF manipulation in ACP + MS treated plots and fertilizer caused marginal declines in colonization in 2,4-D treated plots (S3 Table). In control and picloram plots, both fertilizer (P < 0.001 and P = 0.006, respectively) and fungicide (P < 0.001 and P = 0.035, respectively) reduced colonization potential. In 2023, only herbicide treatment affected colonization (Χ^2 ^= 17.70, d.f. = 3, P < 0.001), with 2,4-D causing increases in colonization relative to the other herbicides (ACP + MS P = 0.002, picloram P = 0.008; Fig 2B), but not the control (S3 Table). Neither AMF manipulation (Χ^2 ^= 0.97, d.f. = 2, P = 0.615) nor its interaction with herbicide (Χ^2 ^= 10.04, d.f. = 6, P = 0.123) were significant, suggesting recovery of AMF abundances or issues detecting colonization changes in older tansy roots.

**Fig 3:**
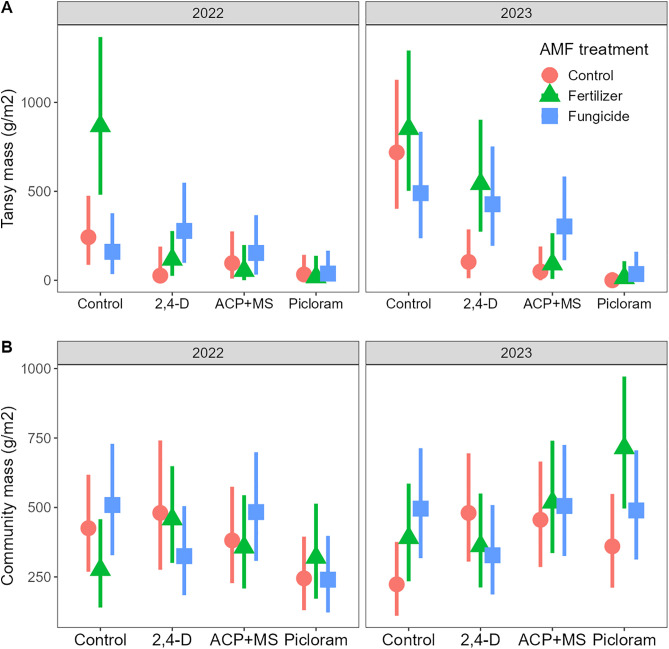
Herbicide and AMF manipulation effects on biomass of *Tanacetum vulgare* (tansy; A) and the remainder of the community (B) in the first (2022) and second (2023) year of the experiment. Herbicide effects differed between years and AMF manipulation treatment effects differed among herbicides for both *Tanacetum vulgare* and the rest of the community (see Table 1 for statistics). Shown are back-transformed model estimated marginal means and 95% confidence intervals.

Consistent with expectations, all three herbicides provided control of common tansy (P < 0.001), although this differed between years (P = 0.036; Table 1). There was no difference among herbicides in 2022 (S3 Table; Fig 3A). In 2023, while all three herbicides continued to reduce tansy biomass (S3 Table), control with 2,4-D and ACP + MS was less than with picloram (P < 0.001 and P = 0.016, respectively) and 2,4-D was marginally less effective than ACP + MS (P = 0.065), indicating recovery consistent with differences in the length of the soil residual effect (Fig 3A).

**Table 1:** Mixed model results showing the effects of herbicides, AMF manipulation, and year on the biomass of tansy and the remainder of the community, the relative cover of grasses, legumes, and other broadleaf plants, and the species richness of the plots.

Factor	Tansy mass			Community mass		
	df	F	P	df	F	P
Herbicide	3,45	29.22	<0.001	3,45	0.84	0.478
AMF	2,49	4.05	0.024	2,53	0.45	0.639
Year	1,49	6.73	0.016	1,49	7.82	0.007
Herbicide:AMF	6,49	3.25	0.009	6,53	2.43	0.036
Herbicide:Year	3,49	3.07	0.036	3,49	8.01	<0.001
AMF:Year	2,52	0.36	0.703	2,50	2.28	0.113
Herbicide:AMF:Year	6,51	1.34	0.259	6,50	1.52	0.191
**Factor**	**Grasses**			**Legumes**		
	**df**	**F**	**P**	**df**	**F**	**P**
Herbicide	3,49	48.82	<0.001	3,44	27.24	<0.001
AMF	2,58	1.01	0.370	2,49	1.47	0.241
Year	1,49	0.08	0.773	1,48	0.01	0.937
Herbicide:AMF	6,58	0.63	0.703	6,50	3.17	0.010
Herbicide:Year	3,48	6.00	0.001	3,48	1.50	0.226
AMF:Year	2,49	3.89	0.027	2,50	0.94	0.398
Herbicide:AMF:Year	6,49	0.89	0.506	6,50	2.22	0.057
**Factor**	**Other broadleaf plants**			**Species richness**		
	**df**	**F**	**P**	**df**	**F**	**P**
Herbicide	3,46	15.02	<0.001	3,45	29.62	<0.001
AMF	2,55	1.90	0.159	2,50	4.97	0.011
Year	1,49	0.21	0.645	1,48	13.67	0.001
Herbicide:AMF	6,55	1.41	0.228	6,50	0.60	0.732
Herbicide:Year	3,49	2.92	0.043	3,48	3.46	0.023
AMF:Year	2,50	0.17	0.840	2,51	0.05	0.953
Herbicide:AMF:Year	6,49	1.48	0.205	6,50	1.59	0.168

All models were run as mixed models with degrees of freedom estimated with Satterthwaite’s method.

Contrary to our hypothesis, both fertilizer and fungicide application increased tansy biomass, but these effects differed among the herbicide treatments (P = 0.009; Table 1). In the control, fertilizer increased tansy growth significantly relative to fungicide (P = 0.005) and marginally relative to the control (P = 0.077), but fungicide did not differ from the control (S3 Table). Surprisingly, with 2,4-D, both fertilizer (P = 0.037) and fungicide (P = 0.015) increased tansy growth relative to the control, suggesting that both AMF manipulation treatments reduced the efficacy of the non-residual herbicide. Consequently, it was only in the AMF suppression plots where 2,4-D offered poorer control than the residual herbicides (S3 Table), whereas we had hypothesized the opposite. Neither AMF manipulation treatment significantly affected tansy biomass within either the ACP + MS or the picloram treatments, potentially due to their enhanced efficacy (S3 Table). Within the fungicide treatment, however, tansy biomass in the ACP + MS was marginally greater in than the picloram treatment (P = 0.064) and no different than the control (S3 Table), suggesting that fungicide may also reduce the efficacy of ACP + MS in addition to reducing 2,4-D efficacy.

Treatment effects on community biomass were also affected by both herbicides and AMF treatments. Herbicide effects were dependent on year (P < 0.001; Table 1). In 2022, picloram reduced community biomass marginally relative to the control (P = 0.098), 2,4-D (P = 0.046), and ACP + MS (P = 0.065), but no other herbicides had any effect (S3 Table). By contrast, in 2023, the only difference was a marginal increase in community biomass with picloram (P = 0.078; Fig 3B), indicating that community biomass recovered from the initial negative effects of picloram. The effect of the AMF treatments again depended on the herbicide (P = 0.036; Table 1). Fungicide increased community biomass marginally relative to both untreated (P = 0.103) and fertilized (P = 0.100) plots in the herbicide control and fertilizer increased community biomass relative to the control in the picloram treatment (P = 0.048), but neither AMF manipulation had an effect in the two other herbicide treatments (S3 Table; Fig 3B).

**Fig 4:**
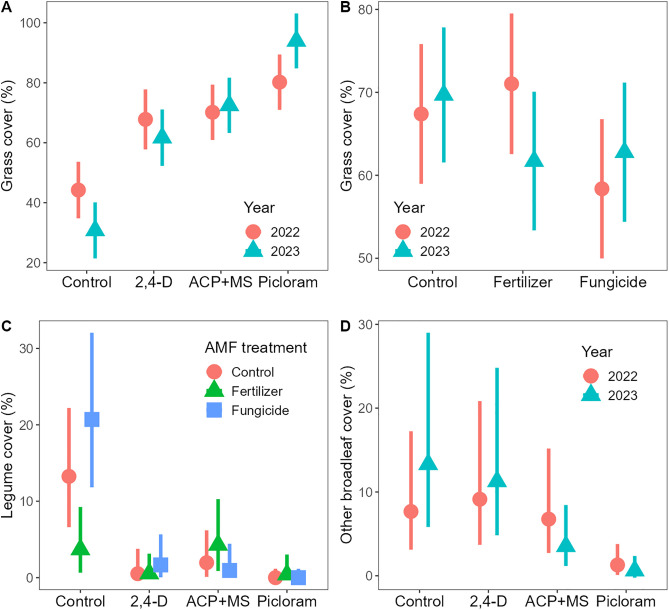
Herbicide, AMF manipulation, and year effects on the relative cover of different plant functional groups. Herbicide (A) and AMF manipulation (B) effects on grass differed significantly between years. AMF treatment effects on legumes differed significantly among herbicides (C), and herbicide effects on other broadleaf plants differed between years (D; see Table 1 for statistics). Shown are back-transformed model estimated marginal means and 95% confidence intervals.

Functional group cover changed with herbicide and AMF treatments, but the responses were functional group specific. Grasses increased in abundance with all herbicides (P < 0.001, S3 Table), indicating release from competition with tansy, but the differences among herbicides varied between years (P = 0.001; Table 1, Fig 4A). The picloram treatment, which was not different from the other herbicides in 2022 (S3 Table), increased grass cover relative to both 2,4-D (P < 0.001) and ACP + MS (P = 0.002) in 2023, likely owing to the stronger suppression of tansy. We also found a significant effect of AMF manipulations that varied over time (P = 0.027; Fig 4B). Contrary to our hypothesis, fertilization had no positive effect on grass cover (S3 Table), whereas fungicide reduced grass cover, but only significantly so relative to the fertilizer treatment and only in 2022 (P = 0.039, S3 Table).

Legumes declined in all herbicide treatments (P < 0.001; S3 Table; Fig 4C), although less so with ACP + MS relative to picloram (P = 0.005), consistent with improved legume retention with this particular herbicide. The AMF manipulation treatments only had an effect when no herbicide was applied (interaction P = 0.002; Table 1), due to herbicide suppression of the legumes. In absence of herbicide, fertilizer decreased legume cover relative to both the control and fungicide (Fig 4C; P = 0.021 and P < 0.001, respectively). As a consequence, there were no significant differences among herbicide treatments when fertilizer was applied (S3 Table), highlighting the strong suppressive effect that nitrogen-based fertilizers can have on nitrogen fixing species.

Cover of other broadleaf plants was also diminished by herbicides (P < 0.001; Table 1), except for 2,4D (Fig 4D), although herbicide effects differed between years (P = 0.043). In 2022, only picloram reduced broadleaf plants relative to the control (P = 0.001; S3 Table), whereas by 2023 both ACP + MS (P = 0.008) and picloram (P < 0.001) had suppressive effects. We found no significant effects of the AMF treatments (Table 1).

**Fig 5:**
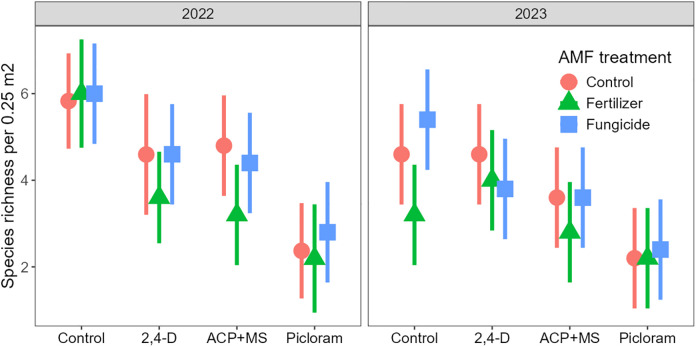
Herbicide and AMF manipulation effects on plant species richness in the first (2022) and second (2023) year of the experiment. Shown are back-transformed model estimated marginal means and 95% confidence intervals. Herbicides effects on richness differed among years, whereas AMF manipulation effects were independent of the other treatments (see Table 1 for statistics).

Plant species richness declined with herbicide application (P < 0.001; Table 1), but the effects differed between years (P = 0.023): Plant species richness declined initially with all herbicides, but especially with picloram, which had stronger effects than both 2,4-D and ACP + MS (both P < 0.001; Fig 5; S3 Table). By the second year, however, richness had recovered in the 2,4-D plots (S3 Table), consistent with our hypothesis. Richness remained intermediate in the ACP + MS plots, which differed from the control (P = 0.038) but not 2,4-D, and lowest in the picloram plots, which was lower than the control (P < 0.001), 2,4-D (P < 0.001), and ACP + MS (P = 0.038). AMF treatments were significant (P = 0.005) and independent of both herbicide and year (Table 1), with fertilizer causing species losses compared to both the control (P = 0.031) and fungicide (P = 0.020; Fig 5).

## Discussion

Contrary to our hypothesis, neither fungicide nor fertilizer improved common tansy control. Instead, both fungicide and fertilizer improved tansy growth, depending on the herbicide treatment. The residual herbicides, specifically picloram, provided the most consistent control, although 2,4-D on its own was nearly equivalent. When applied with either fungicide or fertilizer, however, 2,4-D showed reduced efficacy. Considering the response of the rest of the community, picloram applied with fertilizer caused the greatest increase in grass abundance, but both picloram and fertilizer caused species loss and declines in legume and broadleaf abundances. Picloram based control may thus be most effective but may not be suitable when managing land for conservation. On its own, however, 2,4-D, had no long-term effects on species richness or non-leguminous broadleaf cover, suggesting that 2,4-D may be the safest and most effective treatment for common tansy, at least over the two years of this study.

As evidenced by the reduced colonization potential in 2022, fungicide and fertilizer initially suppressed AMF; however, this did not reduce tansy growth, suggesting that AMF suppression is not a fruitful avenue for tansy control. These results contradict recent suggestions that Asteraceae exploit AMF to invade [19], but are consistent with other studies showing variability in mycorrhizal response within the clade [32,33]. The lack of a response may be because tansy roots were rapidly recolonized after suppression, as suggested by the lack of fungicide or fertilizer effects on tansy root colonization in 2023. AMF communities, however, are often composed of weedy species following disturbance that may be less beneficial [46]. Additionally, given that roots can live multiple years [47] and that the stain binds to chitin whether fungi are dead or alive, it is possible that colonization levels in older roots were overestimated and that fungicide or fertilizer had some effect that we could not detect in 2023. Regardless of whether AMF were effectively suppressed by fungicide or fertilizer, neither appear effective in limiting tansy growth. Indeed, both treatments caused increases in tansy biomass, suggesting that they carry significant risk and are a poor direction to pursue for tansy control.

Improved tansy growth in fungicide treated plots suggests that common tansy suffers from significant pathogen loads in its invaded range. This is consistent with the negative plant-soil feedback previously found for common tansy both in its natural and invaded ranges [17,28]. Feedback on tansy growth was restricted to herbicide treated plots, however, indicating that pathogens may only affect weakened or new tansy plants. Pathogens are most effective at the seedling stage and can also have stronger effects on stressed plants [48,49], suggesting that they may amplify herbicide efficacy.

Rather than suppressing tansy, fertilizer increased tansy biomass, especially when no herbicides were applied. This suggests that tansy is competitive for soil resources relative to the resident species. It also contradicts a hypothesized benefit of fertilizer: to increase grass abundance and thus competitive suppression of invasive broadleaf plants [50]. Indeed, we found no increase in grass cover with fertilizer and the only effect on community biomass occurred after tansy was controlled with picloram. Fertilizer may be effective against other tansy populations, as tansy genotypes differ in their competitive abilities [51]. As we applied both nitrogen and phosphorus fertilizer and, given that plants vary in their ability to mobilize and use different resources [52], other fertilizer formulations may be more effective if tansy differs in its resource requirements from the resident community. It is also possible that fertilizer may have other benefits, including reducing defensive compounds, that may increase consumption of the weed by animals or pathogens [34]. In experimental trials, tansy can decline after strong initial growth in fertile soils [53], which may be attributable to increased pathogen or herbivore pressure. If sufficient to reduce adverse health effects on livestock, this could increase the potential for herbivore control, yet any such inferences are beyond the scope of our results. Nonetheless, we recommend against fertilizer addition in a tansy control program unless future research can identify circumstances or formulations that are safe.

Between the fertilizer and fungicide treatments, fertilizer had the largest effects on the plant community. Species losses to fertilizer addition are well documented [35,54] and our results support this finding, even in seeded pasture. As fertilizer caused significant losses only for legumes, loss of legumes likely led to these declines in species richness, although other broadleaf species may have been lost without causing declines in overall broadleaf cover. These losses occurred even when fertilizer did not cause an increase in community biomass (2,4-D and ACP + MS plots), suggesting that it is not only the competition for light that is causing species losses. Fungicide, conversely, had no effect on most community measures, although previous work has shown that fungicides can alter competition and reproduction, and thus community composition in northern grasslands [23,55,56].

Contrary to our expectation, control of common tansy was similar among the three herbicides after two years, except when other treatments were applied. Higher application rates of 2,4-D, like used here, can control perennial rangeland weeds at least one year after treatment [57]. Consequently, for shorter term control, there is no reason to use residual herbicides. Over longer periods, however, picloram is likely to provide the best control as we saw tansy recovery in 2,4-D and ACP + MS treated plots, which suggests that at least some plants survived application of these herbicides, and is consistent with the varying residual duration among the herbicides [36]. This contrasts with previous work indicating that metsulfuron (MS) can provide better control than picloram [10], suggesting that herbicide efficacy may be site or formulation dependent.

Although picloram provided the best control of tansy, it also had the strongest non-target effects. The negative effect of picloram on grassland biodiversity is well documented, with non-target effects often exceeding those of other residual herbicides [58]. We found an approximately 60% decline in species richness, which is consistent with work from other systems [59]. Biodiversity can recover from picloram application within six years; however, so can the target weed species [59]. Reapplication would thus be required, which can increase non-target herbicide effects [60]. Consequently, picloram application is likely only suitable in forage production systems, where increasing grass biomass is a primary goal. In this scenario, fertilizer may also improve forage production; however, it is unclear whether this would improve tansy suppression over longer time periods. When maintaining biodiversity is a more central focus, either 2,4-D or ACP + MS may be more appropriate. There were no observed negative effects of 2,4-D on species richness after two years, although legume biomass remained low. Although we found no significant effect, aminocyclopyrachlor (ACP) has also been shown to be less detrimental to legumes than some other residual herbicides [aminopyralid; 61], suggesting it may be the better choice in legume heavy systems. Nevertheless, we strongly recommend the use of herbicides that lack residual effects or have shorter residuals when controlling invasive species for conservation purposes.

### Conclusions and future directions

There is a tradeoff between herbicide efficacy and non-target effects as herbicides are never species specific. Consequently, land managers must decide whether they want better weed control or greater biodiversity as herbicides with longer residuals may provide greater control, but also have longer lasting non-target effects. Although alternative means of enhancing control remain attractive, even common methods, like fertilizing the grass stand to make it more competitive, carry significant risks, as we demonstrated for common tansy. More recent ideas, like manipulating the soil biome may have future application; however, both fertilizer and fungicide increased tansy growth and we suggest great caution if attempting similar methods for other invasive species. Nonetheless, it is possible that different fertilizer formulations or other means of manipulating the soil biome (e.g., introduction of pathogens) may be effective in common tansy control. Such ideas, however, would require a more detailed study of the nutrient requirements and pathogen loads of common tansy in its invaded range.

## Supporting information

S1 Appendix All R code used in the analysis. (TXT)

S2 Dataset All data used in the paper.
(CSV)

S3 Table Pairwise comparison results for all models. Each model is represented by a tab in the spreadsheet.
(XLSX)
